# Lens-sparing pars *plicata vitrectomy* for stage 4 retinopathy of prematurity

**DOI:** 10.4103/0301-4738.62661

**Published:** 2010

**Authors:** Parag K Shah, V Narendran, N Kalpana

**Affiliations:** Pediatric Retina and Ocular Oncology Department, Aravind Eye Hospital and Postgraduate Institute of Ophthalmology, Coimbatore, Tamil Nadu, India

Dear Editor,

We read with interest the article by Bhende *et al*.[1] We would like to make the following comments:

1. The title of the article mentions pars plana vitrectomy. We feel that in these young eyes the pars plana is still not developed and the sclerotomies are actually through the pars plicata.
2. Not all cases of Stage 4A retinopathy of prematurity (ROP) require surgery. Some of these remain stable and some get better spontaneously. Only those eyes which are progressing in spite of good laser or unlasered late referrals with vascular activity should be operated upon.
3. It is commendable that in spite of having iatrogenic breaks in three cases, two had a favorable anatomical and visual outcome. In our experience,[2] all the eyes with iatrogenic break did badly. In fact we have even concluded that aggressive peeling in Stage 4B should be avoided for the same reason.
4. With the advent of 23 and 25-gauge systems, lens-sparing vitrectomy (LSV) has become more popular. The small instruments allow the surgeon easy access to anterior membranes in peripheral detachments in these small eyes.[3] However, the sclerotomies should be sutured at the end of the surgery.
5. Triamcinolone acetonide-assisted vitrectomy has been useful in adults. It has also been used in Stage 5 ROP.[4] We are of the opinion that in the future it may become a very useful adjuvant in LSV for Stage 4 ROP too.
